# Supplementary material for: Pachytene piRNAs define a conserved program of meiotic gene regulation: Pachytene piRNAs directly regulate select mRNAs by and RNAi-like mechanism, establishing a regulatory paradigm of mammalian meiosis
Source: Res Sq. 2025 Nov 28:rs.3.rs-8041753. Preprint. [Version 1] doi: 10.21203/rs.3.rs-8041753/v1 (PMC12676428; doi:10.21203/rs.3.rs-8041753/v1)
Supplement: 1 [file NIHPPRS8041753V1-supplement-1.pdf]

METHODS

Animals

Animals C57BL/6 were obtained from Taconic Biosciences and maintained under controlled conditions (12h light/dark cycle, room temperature, and 30-70% humidity, depending on the season) with food and water provided *ad libitum*. Animals were euthanized by CO2 followed by cervical dislocation prior to testis dissection. All animal procedures complied with NIH guidelines and were approved by the NIDDK Animal Care and Use Committee (ACUC) under protocol ASP #K009-LCMB-23.

Generation of *piC-as(Ago2)*, *piC-as(Spin1)* and *piC-as(Spin1)-PGF* knock-out mice

Knockout mice were generated using CRISPR–Cas9–mediated genome editing by electroporation. Pairs of sgRNAs were designed to cleave the predicted promoter regions of *piC-as(Ago2)* (sequences of guide RNAs: sgRNA 5'-UAAAGCCGAAAUGAGAAC-3'; sgRNA 5'-CGGGGUUAGCUGCUUACCCU-3'), *piC-as(Spin1)* (sequences of guide RNAs: sgRNA 5′-GACAUUAAAUGGGGUCGUAG-3′; sgRNA 5′-UAUAGCCUCGUACUCUUUGA-3′) and the *Spin1* pseudogene fragment within the *piC-as(Spin1)* locus on chromosome 9 (sequences of guide RNAs: sgRNA 5'-AGUGGAUGAUACUGUGGCGU-3'; sgRNA 5'-UUAAUAGGCAAAGUAGGGUA-3'). The deletions removed a 646 bp promoter in *piC-as(Spin1)*, a 668 bp promoter in *piC-as(Ago2)* (abolishing piRNA production from these regions; **Fig. 2a, S3a**), and a 1,837 bp region in *piC-as(Spin1)-PGF* mice, removing the *Spin1* pseudogene fragment.

For each construct, two founder animals carrying heterozygous deletions were used to establish independent lines by backcrossing with wildtype C57Bl/6 mice for several generations. Homozygous knockout animals were subsequently obtained through heterozygous intercrosses. Both lines of each construct exhibited identical histological phenotypes; therefore, one representative line per construct was used for downstream analyses.

Genotyping was performed using ear biopsies lysed in PCR-compatible buffer (DEP-25 DNA Extraction Kit, Top-Bio) according to the manufacturer's instructions. PCR products were sequenced by Sanger sequencing to precisely define deletion boundaries. Primer sequences and exact deletion junctions are provided in Source Data 1. Subsequent colony genotyping using real-time PCR and genome-wide genetic monitoring using 10,000 SNP analysis across 241 inbred strains of mice [1] were performed by Transnetyx.

Histology and immunofluorescence

Testes and epididymides were fixed in Hartman's fixative (Sigma-Aldrich) for 1.5 h or overnight at 4°C, dehydrated through an ethanol series, embedded in paraffin, and sectioned at 5 µm. Sections were stained with hematoxylin and eosin or processed for immunofluorescence. Histological images were analyzed using QuPath software [2], and seminiferous tubules were manually examined for germ cell defects (**Fig. 3c, d; Source Data for Fig. 3c,d**).

For immunofluorescence, sections were deparaffinized, subjected to antigen retrieval in 10 mM sodium citrate buffer (pH 6.0) by boiling, and blocked for 45 min in 5% normal donkey serum and 5% bovine serum albumin (BSA) in PBS. Primary antibodies were applied overnight at 4 °C: anti-ɣH2AX (Milipore 05-636, 1:200), anti-ACVR1 (Proteintech 11108-1-AP, 1:200), anti-α-Tubulin (Sigma-Aldrich T6199, 1:100). Alexa-fluor conjugated secondary antibodies (Alexa 488, 594, 647; Thermo Fisher, 1:500) were incubated for 1 h at room temperature. Nuclei were stained with 1 µg ml^{-1} DAPI for 10 min and slides were mounted in ProLong Diamond Antifade Mountant (Thermo Fisher Scientific). Images were acquired using the Zeis 880 confocal microscope and processed in ImageJ [3,4] using identical adjustment parameters.

TUNEL assay

Testes and epididymides were fixed, processed, and sectioned as described above. DNA fragmentation was detected by terminal deoxynucleotidyl transferase (TdT) and labeling with digoxigenin. Images were analyzed in QuPath [2] using identical settings (DAB channel inverted, min = 0.186, max = 0.997, gamma =1). Seminiferous tubules were manually evaluated, and TUNEL-positive tubules were quantified for apoptotic indices.

RNA sequencing

Total RNA was isolated from adult mouse testes using the PureLink miRNA Isolation Kit (Thermo Fisher Scientific) following the manufacturer's instructions, with retention of the long RNA fraction (>200nt), which was further purified with TRIzol. RNA-seq libraries were prepared using the NEBNext Ultra II directional RNA library Prep kit with rRNA depletion and sequenced on Illumina NovaSeq platform (50 bp paired-end reads).

Small RNA-sequencing

Small RNAs were extracted from adult mouse testes using the PureLink miRNA Isolation Kit (Thermo Fisher Scientific) with Trizol according to the manufacturer's instructions. Library preparation followed the general approach of [5] with modifications. Small RNAs were ligated to 3′ indexed adapters containing two unique molecular identifiers (UMIs; **Source Data 1**), and ligation products corresponding to 19- to 32-nt were gel-purified from a 10% urea PAGE. The 3′-ligated RNA was then ligated to 5′ DNA-RNA hybrid adapter containing eight UMIs (**Source**

Data 1), reverse-transcribed, PCR amplified, and size selected using Pippin Prep (3% urea-PAGE). Libraries were sequenced on an Illumina NovaSeq for 50 cycles (single-end reads).

RNA-seq and differential gene expression analysis

RNA-seq reads were mapped to the *Mus musculus* mm10 reference genome using STAR v2.7.10b [6] with up to 100 alignments per read and a maximum mismatch ratio of 0.05. For publicly available RNA-seq data, late-spermatocyte RNA-seq samples GSE41985, MIWI knockout (KO): SRR610676-8 and heterozygous (HET) controls: SRR610673-5 [7] were used. Mapped reads were quantified using featureCounts v.2.0.0 [8]. Protein-coding gene expression was quantified over RefSeq-annotated exons [9], while transposable element expression was calculated using multimapping-aware parameters (*-M -O --fraction*) with UCSC annotation [10]. Pachytene piRNA cluster analysis (considering 90th percentile of top productive pachytene piRNA clusters, **Fig. 2d**, **Source Data for Fig. 2d_top/bottom**) was performed using mouse MIWI cluster annotations (Data S1-mouse MIWI in [11]).

For paired-end libraries, the flags *-p* and *--countReadPairs* were used to count fragments; strandedness was specified with *-s 2*.

Differential gene expression was analyzed in R (https://www.R-project.org/; [12]) using DESeq2 [13] with the model *design ~ condition* (control as reference). Genes with adjusted $p < 0.05$ were considered significantly up- or downregulated (**Fig. 2c**, **Extended Data Fig. 3f**, **Source Data for Fig. 2c_top/bottom** and **Source Data for Extended Data Fig. 3f**).

For subsequent data analysis of MIWI KO and HET mice [7], log2FCs were shrunk with apeglm [14] and empirical cumulative distribution functions (ECDFs) were drawn from shrunken log2FC values. Genes were categorized based on their targeting through pachytene piRNAs: 88 top-target genes (as described below), 9,622 low-target genes (targeted by at least one piRNA but not top-ranked), and 11,799 non-target genes (not targeted by any piRNA). Following DESeq2 filtering of genes with insufficient counts, the final group sizes were 86, 9,288, and 9,497 genes, respectively. Group differences were assessed by two-sample Kolmogorov–Smirnov tests (**Fig. 1d**). Shrunken log2FCs were plotted against −log10(padj) obtained from the unshrunken DESeq2 results (padj and baseMean, **Extended Data Fig. 1c**).

Processing mouse and human small RNA sequencing data.

Mouse small RNA-seq libraries were processed as previously described [15]. In brief, the 3′ adapter sequence (ACGACTTGGAATTCTCGGGTGCCAAGG) was trimmed, and reads were collapsed by sequence to remove PCR duplicates. Unique molecular identifiers (UMIs, 8 nt at the 5' end and 2 nt at the 3' end) were then excised. Adapter and UMIs were removed using

Cutadapt v4.4 [16]. Reads shorter than 20 nt were discarded. Structural RNAs were removed prior to genomic alignment. Remaining reads were mapped to the mouse genome (mm10) with splicing disabled, allowing up to 100 alignments per read and a maximum of one mismatch per alignment, using STAR v2.7.10b [6]. FASTQ quality was assessed with FastQC v0.11.9 (https://www.bioinformatics.babraham.ac.uk/projects/fastqc/). BAM files were sorted and indexed using Samtools v1.17 [17].

For the human analysis, published small RNA-seq of an adult sample (SRR8575350, [18]) was processed as previously described [11].

Unless otherwise stated, all subsequent computational analyses were performed in R v4.4.2 (https://www.R-project.org/; [12]) using CRAN and Bioconductor [19] packages. PiRNA alignments were imported into the R environment using the PICBload function from PICB [15] with BSgenomes (mouse: BSgenome.Mmusculus.UCSC.mm10 and human: BSgenome.Hsapiens.UCSC.hg38) [20]; *GET.ORIGINAL.SEQUENCE = TRUE*, and otherwise default parameters. Published mouse PIWIL2 (MILI) and PIWIL1 (MIWI) pachytene, and human adult piRNA clusters [11] were used for downstream analyses and ranked by productivity (mouse: PICB column '*all_reads_primary_alignments_FPM*'; human: *'reads_explained'*).

Establish potential direct targets of pachytene piRNAs

To determine the direct targets of mouse and human pachytene piRNAs within protein-coding genes (PCGs), custom transcriptomes of protein coding genes were generated. Gene annotations were retrieved from NCBI RefSeq for mouse (mm10; [9]) and from Gencode for human (GRCh38.p14, v44 primary assembly; [21]). Corresponding genomic sequences were obtained from BSgenome packages (BSgenome.Mmusculus.UCSC.mm10 or BSgenome.Hsapiens.UCSC.hg38, [20]).

Predicted transcripts (XM_* and XR_*) were excluded. Overlapping exons were collapsed and for each gene, overlapping isoforms were merged into nonredundant genomic intervals retaining the annotation hierarchy CDS > 3′UTR > 5′UTR.

To enable direct mapping, the custom transcriptome consists of the collapsed gene sequences with 2 kb "N" buffers to avoid cross-gene alignments in FASTA format and a corresponding GTF file containing both transcriptome and original chromosomal coordinates. Primary alignments previously mapped to the mm10 or hg38 genomes (as described above) were selected and remapped to the custom transcriptome using STAR [6] with splicing disabled. To model conservative piRNA targeting, reads were aligned stringently by enforcing contiguous base pairing from positions 2–21 through 5′ extension while excluding the first nucleotide *(--alignEndsType Extend5pOfRead1; --clip5pNbases 1*) and requiring a minimum of 19 matched bases *(--outFilterMatchNmin 19*). A single mismatch (*--outFilterMismatchNmax 1*) and up to

100 multimapping loci per read and anchor (*--winAnchorMultimapNmax 100 --outFilterMultimapNmax 100*) were permitted.

For target identification, uniquely mapping piRNAs aligning antisense to protein-coding genes in the custom transcriptome were included. Genes targeted predominantly by piRNAs originating from a MILI pachytene piRNA cluster located antisense to the same gene were excluded from the analysis (mouse: 157, human: 697 genes). Targeted genes were ranked by cumulative antisense piRNA counts (**Fig. 1a, 4a**). Detailed results for both mouse and human datasets are provided in **Source Data for Fig. 1 and 4**, including targeting rank, total piRNA counts, rank of the top contributing piRNA cluster, and other target-specific piRNA clusters metrics used in **Fig. 1 and 4**. Pseudogene annotations were obtained from the UCSC Table Browser (mouse, [10]) and the Gencode pseudogene annotation (human, v47, [21], *GOLGA2P11* was manually added based on UCSC annotation).

To demonstrate the piRNA coverage over genomic loci, piRNAs were separated by strand, normalized to reads-per-million (rpm) per tile (1-nt tiles for **Fig. 1b,f,g** and **Extended Data Fig. 1b**; 50-nt tiles for **Extended Data Fig. 1e,f**: 100-nt tiles for **Fig. 2a** and **Extended Data Fig. 3a**), and visualized as strand-resolved coverage profiles. To show targeting across the full *Spin1* and *Ago2* genomic regions, piRNA reads were remapped to locus-specific mm10 genomic references (+/- 50 nt around each gene boundary, *Spin1*: chr13: 51100830-51152612; *Ago2*: chr15: 73101575-73184997) using the same parameters described for mapping against the custom transcriptome. After remapping, only uniquely aligned reads to both the locus and the mm10 genome were retained, and coverage values were normalized relative to all uniquely mapped reads in the respective gene regions (**Extended Data Fig. 1e,f**).

The distribution of mRNA-targeting piRNAs across transcript features was determined by partitioning each feature (5′UTR, CDS, 3′UTR) in the custom protein coding transcriptome into 20 equal-length bins per gene, maintaining strand orientation. The number of piRNA 5′ ends mapping antisense to each bin was counted, normalized to total gene-targeting piRNAs, and expressed as percentages. Bin-wise averages across all genes yielded the positional metagene profile. In both mouse and human, three genes were excluded from the analyses due to too short 5'UTRs (< 20 nt, **Extended Data Fig. 1a** and **Extended Data Fig. 4a**). Targeting-coverage lengths were computed per gene as the summed base pairs of overlap between collapsed piRNA alignments antisense to the gene (**Fig. 1c, 4b**).

To trace the origin of mRNA-targeting piRNAs, antisense alignments to the top 88 mouse and 50 human gene targets (hereafter referred to as “top-targeted genes”) were grouped by their cluster of origin. Read contributions from individual pachytene piRNA clusters were quantified and normalized to total gene-targeting piRNA counts. Clusters contributing less than 5% of reads to the targeting of the individual gene were pooled as “piCs < 5%” and non-cluster-derived reads were reported separately (“non-piC-derived”). The resulting fractions were visualized as stacked

bar charts (**Extended Data Fig. 1d and S4d**). To highlight the dominant source per gene, the single top-contributing piC fraction was plotted against gene rank (**Fig. 1e, Fig. 4b**). For all piRNAs targeting mRNAs in trans (81.02% of total and 96.49% among top-targeted genes in mouse; 59.61% of total and 95.45% among top-targeted genes in human), the fraction originating from pachytene piRNA clusters was calculated (82.38% of total and 85.94% among top-targeted genes in mouse, 64.66% of total and 81.72% among top-targeted genes in human, **Insets in Fig. 1e and 4b** show fractions of top-targeted genes).

Sequence-identity and synteny analysis

To compare the *Spin1* and *Ago2* pseudogene and gene sequences, the sequence and annotations of both the pseudogene region (*Spin1*: chr9:67747306-67748766; *Ago2*: chr4:123838102-123839732) and the genes' corresponding exon and intron regions (*Spin1*: chr13:1698750-1700210, corresponding to the exonic region targeted by piRNAs with extension to match length of pseudogene region; *Ago2*: chr15:73106583-73123456, exons 11-18, intron between exon 18 and exon 19 and the 5'end of exon 19) were aligned with minimap2 (*-x asm10, -c, --eqx*, secondary alignments suppressed, [22]. The resulting PAFs were parsed with SVbyEye [23] to visualize the identity-colored alignments (binsize=10, **Fig. 1f, g**).

To identify syntenic piRNA clusters around the *C2cd4b-Tln2* locus, gene and pseudogene annotations were retrieved from the UCSC Genome Browser for rhesus macaque (rheMac8), marmoset (calJac3), cow (bosTau8), and rat (rn6); their piRNA cluster coordinates were taken from published annotations (rhesus macaque: *pi_rheMac8_IG_99.1* and *pi_rheMac8_PC_C2CD4B.1*; marmoset: *pi_calJac3_PC_C2CD4B.1* and *pi_calJac3_IG_21.1*; cow: *pi_bosTau8_IG_3.1* and *pi_bosTau8_IG_4.1*; rat: *pi_rn6_IG_101.1* and *pi_rn6_IG_102.1*; [24]). Human (hg38) and mouse (mm10) annotations and piCs were obtained as described above. For clarity, only the genes *C2CD4B*, *TLN2*, and the respective *Spin1*/*GOLGA2* pseudogenes are shown (**Fig. 4d**). To quantify sequence conservation between primate loci, the human *GOLGA2* gene, the human *piC-as(GOLGA2)* region, the rhesus *pi_rheMac8_IG_99.1*, the marmoset *pi_calJac3_IG_21.1* [24], and the marmoset *GOLGA2* gene sequences were extracted. All-vs-all alignments were performed with minimap2 (*-x asm20 -c --eqx -D -P --dual=no*; [22]). PAF outputs were parsed and visualized with SVbyEye (binsize=100; [23]) as arc plots, coloring alignments by percent identity (<70, 70–80, 80–90, >90).

***piC-as(Spin1-PGF)* evolutionary analysis**

To determine when the *Spin1* pseudogene fragment inserted into the *piC-as(Spin1-PGF)* cluster, the *Mus musculus* (GRCm38/mm10; GCF_000001635.20) DNA sequence for the pseudogene fragment (chr9:67,747,306-67,748,766) plus 100 base pairs (bp) of flanking sequence were extracted. To identify target site duplications (TSDs) that are characteristic of LINE1-mediated retrotransposition [25], the *Spin1-PGF* plus flanking sequence was aligned to itself using a

permissive blastn search (NCBI blastn, word size = 7, expect threshold 1000), and searched for identical DNA sequence (2-20 bps) flanking the boundaries of *Spin1-PGF*. Once the boundaries of *Spin1-PGF*, including the TSDs, were determined, the *Spin1-PGF* plus flanking sequence were used as a query to identify other species with a syntenic insertion between the *Tln2* and *C2cd4b* genes (NCBI blastn, wgs database, limited to Rodentia: taxid 9989). From this search, additional representative Spin1-PGF sequences (*Mus Pahari* [GCF_900095145.1], *Mus caroli* [GCF_900094665.2], *Mus spicilegus* [GCA_003336285.2], *Mus spretus* [GCA_001624865.1]), or corresponding empty site (ie: single target site, no Spin1-PGF) (*Mastomys coucha* [GCF_008632895.1], *Grammomys surdaster* [SRMG01000153.1], *Rattus norvegicus* [GRCr8; GCF_036323735.1]) were collected (**Supplemental File 1**). The sequences were then aligned (Clustal Omega, default parameters [26] and manually curated [27]. Using the manually curated alignments, the *Spin1-PGF* retrogene/empty sites were annotated (including TSDs, polyA tails, *Spin1* sequence, and flanking sequences; **Supplemental File 2**), and the approximate emergence time for the *Spin1-PGF* using parsimony was determined (**Extended Data Fig. 4e**).

piC-as(GOLGA2-PGF) evolutionary analysis

To determine when the GOLGA2-PGF was acquired by the *piC-as(GOLGA2-PGF)* cluster, the pseudogene fragment (GRCh38.p14/hg38 [GCF_000001405.40] chr15:62,241,721-62,257,703) was extracted and a BLAT [28] search was performed against all genomes deposited in the UCSC genome browser (default parameters) to identify other species with a syntenic GOLGA-PGF (~20kb) between the TLN2 and C2CD4B genes. Additionally, the genomic region between the TLN2 and C2CD4B genes was extracted for three additional species (*Mesocricetus auratus, Jaculus jaculus, and Castor canadensis*) to increase representation for rodent lineages, and a pairwise blastn search (default parameters) was performed between these regions and the human GOLGA-PGF sequence. To identify the percent sequence conservation of the GOLGA-PGF, multiple sequence alignment for the GOLGA2-PGF region plus flanking (chr15:62237763-62260023) were downloaded (UCSC Primate 30way multiz genome-wide alignment) and converted from MAF to multi FASTA using the maf_to_fasta_concat.py tool from Galaxy [29]. The data were filtered to remove non-primates (**Supplemental File 3**).

Model and animal cartoons were created with BioRender (**Fig. 4d,e, Extended Data Fig. 4e**, (BioRender.com)).

Data availability

Sequencing data generated in this study have been deposited at GEO (accession number) and are publicly available as of the date of publication. This manuscript analyzed publicly available data deposited at SRA under accession numbers SRR610676-8, SRR610673-5 and SRR8575350.

Mouse models generated in this study are available at Jackson Laboratory (link).

Any additional information required to reanalyze the data reported in this paper is available from the lead contact upon request.

Code availability

All scripts to computational analyses are available on GitHub (https://github.com/HaaseLab) and in the Source Data Code.

REFERENCES

1 Sigmon, J. S. *et al.* Content and Performance of the MiniMUGA Genotyping Array: A New Tool To Improve Rigor and Reproducibility in Mouse Research. *Genetics* **216**, 905-930 (2020). https://doi.org/10.1534/genetics.120.303596

2 Bankhead, P. *et al.* QuPath: Open source software for digital pathology image analysis. *Sci Rep* **7**, 16878 (2017). https://doi.org/10.1038/s41598-017-17204-5

3 Schindelin, J. *et al.* Fiji: an open-source platform for biological-image analysis. *Nat Methods* **9**, 676-682 (2012). https://doi.org/10.1038/nmeth.2019

4 Schneider, C. A., Rasband, W. S. & Eliceiri, K. W. NIH Image to ImageJ: 25 years of image analysis. *Nat Methods* **9**, 671-675 (2012). https://doi.org/10.1038/nmeth.2089

5 Hafner, M. *et al.* Barcoded cDNA library preparation for small RNA profiling by next-generation sequencing. *Methods* **58**, 164-170 (2012). https://doi.org/10.1016/j.ymeth.2012.07.030

6 Dobin, A. *et al.* STAR: ultrafast universal RNA-seq aligner. *Bioinformatics* **29**, 15-21 (2013). https://doi.org/10.1093/bioinformatics/bts635

7 Watanabe, T., Cheng, E. C., Zhong, M. & Lin, H. Retrotransposons and pseudogenes regulate mRNAs and lncRNAs via the piRNA pathway in the germline. *Genome Res* **25**, 368-380 (2015). https://doi.org/10.1101/gr.180802.114

8 Liao, Y., Smyth, G. K. & Shi, W. featureCounts: an efficient general purpose program for assigning sequence reads to genomic features. *Bioinformatics* **30**, 923-930 (2014). https://doi.org/10.1093/bioinformatics/btt656

9 McGarvey, K. M. *et al.* Mouse genome annotation by the RefSeq project. *Mamm Genome* **26**, 379-390 (2015). https://doi.org/10.1007/s00335-015-9585-8

10 Perez, G. *et al.* The UCSC Genome Browser database: 2025 update. *Nucleic Acids Res* **53**, D1243-D1249 (2025). https://doi.org/10.1093/nar/gkae974

11 Konstantinidou, P. *et al.* A comparative roadmap of PIWI-interacting RNAs across seven species reveals insights into de novo piRNA-precursor formation in mammals. *Cell Rep* **43**, 114777 (2024). https://doi.org/10.1016/j.celrep.2024.114777

12 R: A Language and Environment for Statistical Computing (Foundation for Statistical Computing) (2013).

13 Love, M. I., Huber, W. & Anders, S. Moderated estimation of fold change and dispersion for RNA-seq data with DESeq2. *Genome Biol* **15**, 550 (2014). https://doi.org/10.1186/s13059-014-0550-8

14 Zhu, A., Ibrahim, J. G. & Love, M. I. Heavy-tailed prior distributions for sequence count data: removing the noise and preserving large differences. *Bioinformatics* **35**, 2084-2092 (2019). https://doi.org/10.1093/bioinformatics/bty895

15 Ahrend, F. *et al.* Protocol for assembling, prioritizing, and characterizing piRNA clusters using the piRNA Cluster Builder. *STAR Protoc* **6**, 103759 (2025). https://doi.org/10.1016/j.xpro.2025.103759

16 Martin, M. Cutadapt removes adapter sequences from high-throughput sequencing reads. *2011* **17**, 3 (2011). https://doi.org/10.14806/ej.17.1.200

17 Li, H. *et al.* The Sequence Alignment/Map format and SAMtools. *Bioinformatics* **25**, 2078-2079 (2009). https://doi.org/10.1093/bioinformatics/btp352

18 Ozata, D. M. *et al.* Evolutionarily conserved pachytene piRNA loci are highly divergent among modern humans. *Nat Ecol Evol* **4**, 156-168 (2020). https://doi.org/10.1038/s41559-019-1065-1

19 Gentleman, R. C. *et al.* Bioconductor: open software development for computational biology and bioinformatics. *Genome Biol* **5**, R80 (2004). https://doi.org/10.1186/gb-2004-5-10-r80

20 BSgenome: Software infrastructure for efficient representation of full genomes and their SNPs v. R package version 1.76.0 (2025).

21 Mudge, J. M. *et al.* GENCODE 2025: reference gene annotation for human and mouse. *Nucleic Acids Res* **53**, D966-D975 (2025). https://doi.org/10.1093/nar/gkae1078

22 Li, H. Minimap2: pairwise alignment for nucleotide sequences. *Bioinformatics* **34**, 3094-3100 (2018). https://doi.org/10.1093/bioinformatics/bty191

23 Porubsky, D. *et al.* SVbyEye: a visual tool to characterize structural variation among whole-genome assemblies. *Bioinformatics* **41** (2025). https://doi.org/10.1093/bioinformatics/btaf332

24 Yu, T. *et al.* Long first exons and epigenetic marks distinguish conserved pachytene piRNA clusters from other mammalian genes. *Nat Commun* **12**, 73 (2021). https://doi.org/10.1038/s41467-020-20345-3

25 Kaessmann, H., Vinckenbosch, N. & Long, M. RNA-based gene duplication: mechanistic and evolutionary insights. *Nat Rev Genet* **10**, 19-31 (2009). https://doi.org/10.1038/nrg2487

26 Madeira, F. *et al.* The EMBL-EBI Job Dispatcher sequence analysis tools framework in 2024. *Nucleic Acids Res* **52**, W521-W525 (2024). https://doi.org/10.1093/nar/gkae241

27 Waterhouse, A. M., Procter, J. B., Martin, D. M., Clamp, M. & Barton, G. J. Jalview Version 2--a multiple sequence alignment editor and analysis workbench. *Bioinformatics* **25**, 1189-1191 (2009). https://doi.org/10.1093/bioinformatics/btp033

28 Kent, W. J. *et al.* The human genome browser at UCSC. *Genome Res* **12**, 996-1006 (2002). https://doi.org/10.1101/gr.229102

29 Blankenberg, D., Taylor, J., Nekrutenko, A. & Galaxy, T. Making whole genome multiple alignments usable for biologists. *Bioinformatics* **27**, 2426-2428 (2011). https://doi.org/10.1093/bioinformatics/btr398
